# Risk of Nonarteritic Anterior Ischemic Optic Neuropathy Following End-Stage Renal Disease

**DOI:** 10.1097/MD.0000000000003174

**Published:** 2016-03-25

**Authors:** Yuh-Shin Chang, Shih-Feng Weng, Chun Chang, Jhi-Joung Wang, Shih-Bin Su, Chien-Cheng Huang, Jiu-Yao Wang, Ren-Long Jan

**Affiliations:** From the Department of Ophthalmology (YSC), Department of Medical Research (JJW), Department of Anesthesiology (JJW), Department of Occupational Medicine, Chi Mei Medical Center (SBS), Graduate Institute of Medical Science, College of Health Science, Chang Jung Christian University (YSC), Department of Child Care and Education, Southern Taiwan University of Science and Technology (CCH), Graduate Institute of Clinical Medicine, National Cheng Kung University (JYW, RLJ), Department of Pediatrics, Chi Mei Medical Center, Liouying, Tainan (RLJ), Department of Leisure, Recreation, and Tourism Management, Tainan (SBS, CCH), Department of Healthcare Administration and Medical Informatics, Kaohsiung Medical University, Kaohsiung (SFW), and Department of Education, University of Taipei, Taipei, Taiwan (CC).

## Abstract

To investigate the risk of nonarteritic anterior ischemic optic neuropathy (NAION) following end-stage renal disease (ESRD).

A retrospective, nationwide, matched cohort study.

ESRD patients identified by the International Classification of Diseases, Ninth Revision, Clinical Modification (ICD-9-CM) code 585.

The study cohort included 93,804 ESRD patients registered with the Taiwan National Health Insurance Research Database between January 2000 and December 2009. An age- and sex-matched control group comprised 93,804 patients (case:control = 1:1) selected from the Taiwan Longitudinal Health Insurance Database 2000. Information for each patient was collected from the index date until December 2011. The incidence and risk of NAION were compared between the ESRD and control groups. The adjusted hazard ratio (HR) for NAION after adjustment for potential confounders was obtained by a Cox proportional hazard regression analysis. A Kaplan–Meier analysis was used to calculate the cumulative incidence rate of NAION.

The incidence of NAION following ESRD.

In total, 133 ESRD patients (0.14%) and 51 controls (0.05%) had NAION (*P* < 0.001) during the follow-up period, leading to a significantly elevated risk of NAION in the ESRD patients compared with the controls (incidence rate ratio = 3.14, 95% confidence interval [CI] = 2.11–4.67). After adjustment for potential confounders including diabetes mellitus, hypertension, hypotension, hyperlipidemia, and 2-way interaction terms between any 2 factors, ESRD patients were 3.12 times more likely to develop NAION than non-ESRD patients in the full cohort (adjusted HR = 3.12, 95% CI = 2.10–4.64). Additionally, patients with hypertension and hyperlipidemia showed higher incidence rates of NAION in the ESRD group compared with the controls: 2.31 (95% CI = 1.40–3.82) for hypertension and 2.72 (95% CI = 1.14–6.50) for hyperlipidemia.

ESRD increased the risk of NAION, which is an interdisciplinary emergency. Close collaboration between nephrologists and ophthalmologists is important in NAION management following ESRD to prevent fellow eye involvement.

## INTRODUCTION

End-stage renal disease (ESRD), the most severe form and the last stage of chronic kidney disease, requires dialysis or transplant treatment. ESRD is an important public health issue and a leading cause of morbidity and mortality worldwide. Recently, the prevalence and incidence of ESRD have increased rapidly, not only in Western and Asian populations, but also in developing and developed countries worldwide.^1,2^ Taiwan has demonstrated a particularly high prevalence and incidence of ESRD compared with other countries.^3–5^

Nonarteritic anterior ischemic optic neuropathy (NAION) is the most frequent acute optic neuropathy and a relatively common cause of irreversible vision loss among middle-aged and elderly populations.^6–8^ The diagnosis of NAION is primarily clinical and depends on the manifestation of sudden, painless, monocular vision loss with an accompanying relative afferent pupillary defect and edema of the optic disc.^7,8^ NAION results from hypoperfusion of the prelaminar optic nerve because of reduced flow in the posterior ciliary artery, which supplies the optic nerve head.^6,7^ The pathogenesis of NAION primarily involves hypoperfusion or nonperfusion of the optic nerve head resulting from a small and crowded disc, decreased blood delivery, low blood oxygen carrying capacity, or increased vascular resistance.^6,7,9,10^ A variety of factors can cause the mechanism such as a disc at risk, systemic hypotension, anemia, atherosclerosis, and hypercoagulable states.^9–11^ The risk of sequential involvement of the fellow eye ranges from 12% to 15% at 5 years.^12^

Episodes of hypotension during hemodialysis are serious complications and one of the most common problems affecting ESRD patients.^13–16^ Besides hypotension, anemia is another consequence of hemodialysis that has long been associated with ESRD.^17–20^ In fact, ESRD patients tend to exhibit decreased blood delivery because of intradialytic hypotension, and are prone to low blood oxygen carrying capacity related to anemia. Additionally, ESRD patients are at a higher risk for increased vascular resistance, which is usually associated with atherosclerosis,^21,22^ and hypercoagulation disorders associated with platelet dysfunction and uremic toxin retention.^23,24^ In addition to the apparently common pathogenic mechanisms, ESRD and NAION share common systemic risk factors, including hypertension, hypotension, diabetes mellitus, and hyperlipidemia. Therefore, it is clinically relevant to determine whether ESRD is a predictor of NAION.

Several previous studies have discussed the association between ESRD and NAION, but the results of the published studies were limited by the small number of patients and absence of comparative control data. Using a nationwide, population-based dataset, we designed a cohort study to investigate the risk of NAION following ESRD in Taiwan.

## METHODS

### Database

On March 1, 1995, Taiwan launched a single-payer National Health Insurance (NHI) scheme, which provides extensive medical care coverage for all residents of Taiwan. As of 2007, 22.60 million individuals (>98%) of the total Taiwanese population of 22.96 million were enrolled in this program. The data in our cohort study were obtained from the Taiwan National Health Insurance Research Database (NHIRD). The NHIRD supplies encrypted patient identification numbers as well as information regarding patient sex, birth date, and admission and discharge dates. It also includes the International Classification of Diseases, Ninth Revision, Clinical Modification (ICD-9-CM) diagnoses and procedure codes, prescription details, and the costs covered and paid by NHI. A public database was used for the analysis; therefore, the requirements for ethical approval and informed consent were waived by the Institutional Review Board of Chi-Mei Medical Center, Tainan, Taiwan. The requirement for informed consent was waived because the analyzed datasets were obtained from a database devoid of personally identifiable information.

### Study Design

This retrospective, nationwide, matched cohort study involved 2 groups of participants: a new-onset ESRD group and a matched non-ESRD (control) group.

### Study Participants

Patients and controls were recruited from 2000 to 2009. We included 93,804 ESRD patients who started their first dialysis treatment after December 31, 2000 and who had received a catastrophic illness certificate (CIC) with code number 585 between January 1, 2000 and December 31, 2009. Patients of unknown sex and with missing data were excluded. Patients diagnosed as having NAION (ION [ICD-9-CM code 377.41] without giant cell arteritis [ICD-9-CM code 446.5]) before ESRD were also excluded.

For each ESRD case, one control without ESRD was randomly selected from the Longitudinal Health Insurance Database 2000 (LHID2000), a data subset of the National Health Insurance Research database (NHIRD) that contains full claim data for 1 million beneficiaries (4.34% of the total population) systemic-randomly selected in 2000. There was no significant difference in age, sex, or healthcare costs between the sample group and all NHI enrollees. The 93,804 controls were matched by sex, age, and index date. The index date for the ESRD patients was the date of their first dialysis, and the index date for the controls was created by matching the date with the ESRD subject's index date. Moreover, controls diagnosed with NAION before the index date were also excluded. Each patient was followed until the end of 2011 to determine the incidence of NAION or censored because of death.

To distinguish all patients who had developed NAION (ION [ICD-9-CM code 377.41] without giant cell ∗∗∗arteritis [ICD-9-CM code 446.5]), we tracked every patient from his or her index outpatient visit or hospitalization through December 2011. Demographic data (eg, age and sex) were recorded. Furthermore, we collected information on comorbidities including diabetes mellitus (ICD-9-CM code 250), hypertension (ICD-9-CM codes 401–405), hypotension (ICD-9-CM code 458), and hyperlipidemia (ICD-9-CM code 272) because these conditions are critical factors that increase the risk of NAION. In this study, the inclusion criteria for diabetes mellitus, hypertension, hypotension, and hyperlipidemia were documentation of the condition at least once in an inpatient setting or ≥3 times in an ambulatory setting within 1 year before initial ESRD on the dialysis medical service date.

### Statistical Analysis

SAS 9.4 for Windows (SAS Institute, Inc., Cary, NC) was used in this study. The demographic characteristics and comorbid disorders between the ESRD and control groups were compared by Pearson Chi-squared test. The incidence rate was calculated as the number of NAION cases identified during follow-up divided by the total number of person-years (PY) for each group by age, sex, and select comorbidities. A Poisson regression analysis was performed to calculate the incidence rate ratio (IRR), which demonstrated a comparison of the risk of developing NAION between the ESRD and control groups. The adjusted hazard ratio (HR) for developing NAION was calculated using a Cox proportional hazard regression analysis. Two-way interactions was added to the models for IRR and adjusted HR. Cumulative incidence rates for NAION in ESRD were evaluated by a Kaplan–Meier analysis, and differences in cumulative incidence rate curves were analyzed using the log-rank test. Additionally, we subdivided the patients into 3 age subgroups for further analysis: <50 years, 50 to 64 years, and ≥65 years. Data are presented as the mean ± standard deviation (SD), and 95% confidence intervals (CIs) are provided when applicable. Statistical significance was defined as *P* < 0.05.

## RESULTS

### Demographic Data

Between 2000 and 2009, 93,804 ESRD patients and 93,804 controls were recruited after excluding ineligible subjects. Table 1 provides the demographic characteristics and comorbid disorders of the ESRD patients and the age- and sex-matched controls. The mean age of all participants was 62.22 ± 14.65 years. ESRD patients exhibited a significantly higher prevalence of previously reported comorbidities than the controls, such as diabetes mellitus, hypertension, hypotension, and hyperlipidemia. The mean follow-up periods for the ESRD and control patients were 4.71 (SD, 3.26) and 6.51 (SD, 2.96) years, respectively.

**TABLE 1:**
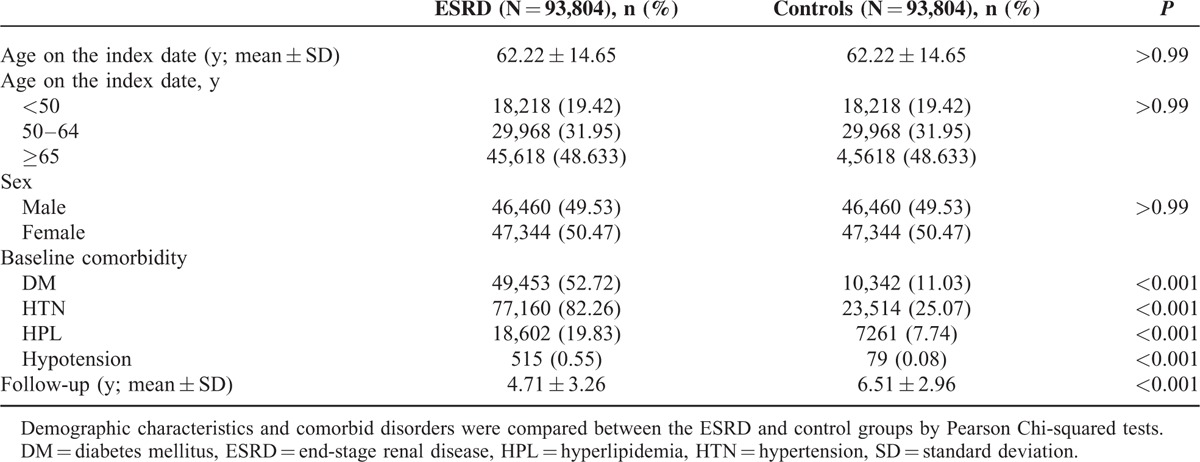
Demographic Characteristics and Comorbid Disorder Comparisons Between the End-Stage Renal Disease and Control Groups

### Incidence Rates of NAION

During the follow-up period, 184 (184/187,608 [0.10%]) patients developed NAION. A significantly higher proportion of ESRD patients (133/93,804 [0.14%]) developed NAION than control patients (51/93,804 [0.05%]) (Table 2). In addition, there was a significant difference in NAION incidence between the groups (ESRD patients = 3.01/10,000 PY; control = 0.84/10,000 PY), and the IRR between the ESRD group and the control group was statistically significant (3.14, 95% CI = 2.11–4.67, *P* < 0.001; Table 2).

**TABLE 2:**
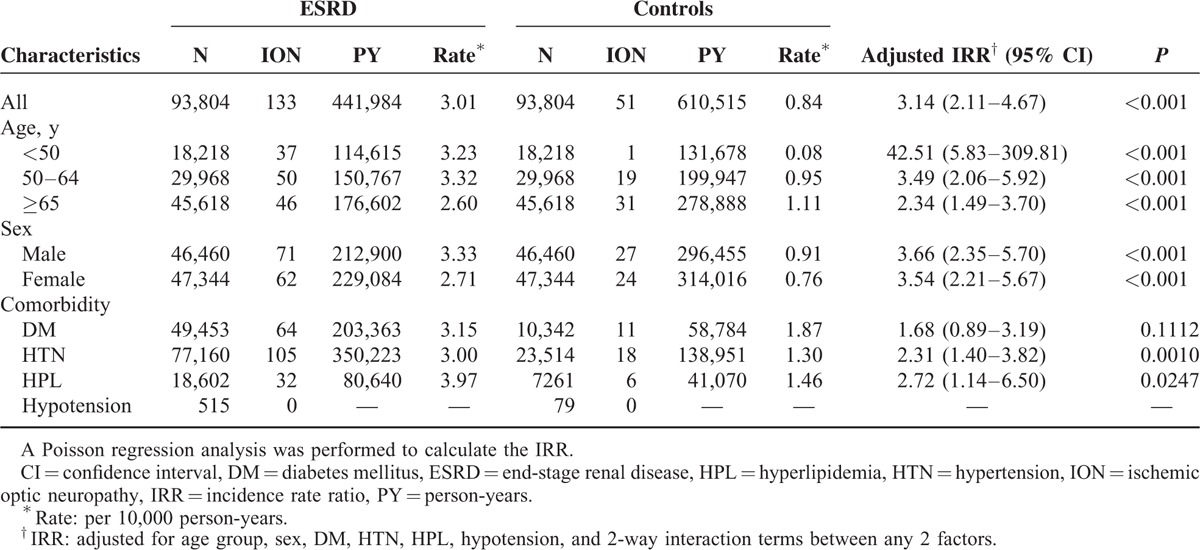
Risk of Ischemic Optic Neuropathy in the End-Stage Renal Disease and Control Groups

After the 2 groups were stratified by age, we found that ESRD patients 50 to 64 years old had the highest incidence rate (3.32/10,000 PY), followed by patients <50 years and patients ≥65 years old. We found significantly higher IRRs for all ESRD age groups compared with their age-matched controls (Table 2).

Male ESRD patients had an NAION incidence of 3.33/10,000 PY, whereas male control group patients had an NAION incidence of only 0.91/10,000 PY, leading to a significant IRR between male ESRD patients and their controls (IRR = 3.66, 95% CI = 2.35–5.70, *P* < 0.001). Regarding female patients, a significant difference in NAION incidence rate was also noted between female ESRD patients and their controls (IRR = 3.54, 95% CI = 2.21–5.67, *P* < 0.001; Table 2).

In the ESRD group, the incidence rates of NAION from highest to lowest were found in patients with hyperlipidemia (3.97/10,000 PY), followed by diabetes mellitus (3.15/10,000 PY) and hypertension (3.00/10,000 PY). There was no incidence of NAION among patients with hypotension in the ESRD group. The IRR for NAION associated with comorbid hypertension and hyperlipidemia indicated significantly greater risks in ESRD patients with these conditions compared with their controls: 2.31 (95% CI = 1.40–3.82) for hypertension and 2.72 (95% CI = 1.14–6.50) for hyperlipidemia (Table 2); however, this was not observed for the presence of diabetes mellitus. We were unable to evaluate whether hypotension in ESRD patients increases the risk of NAION because of the lack of NAION incidence among hypotensive patients in both groups.

Table 3 provides the crude and adjusted HRs for NAION by cohort during the follow-up period. After adjusting for age, sex, select comorbid conditions, and 2-way interaction terms between any 2 factors, ESRD remained an independent risk factor for NAION (adjusted HR = 3.12, 95% CI = 2.10–4.64). Some comorbidities were significant risk factors for NAION in both groups, including diabetes mellitus (crude HR = 1.98, 95% CI = 1.47–2.66, *P* < 0.05), hypertension (crude HR = 2.23, 95% CI = 1.64–3.04, *P* < 0.05), and hyperlipidemia (crude HR = 1.91, 95% CI = 1.33–2.73, *P* < 0.05), but were not significantly greater independent risk factors for NAION after adjusting for age, sex, select comorbid conditions, and 2-way interaction terms between any 2 factors. We could not evaluate whether hypotension was a significant risk factor after adjusting for other confounding factors in the total cohort because of the lack of NAION incidence among hypotensive patients in both groups.

**TABLE 3:**
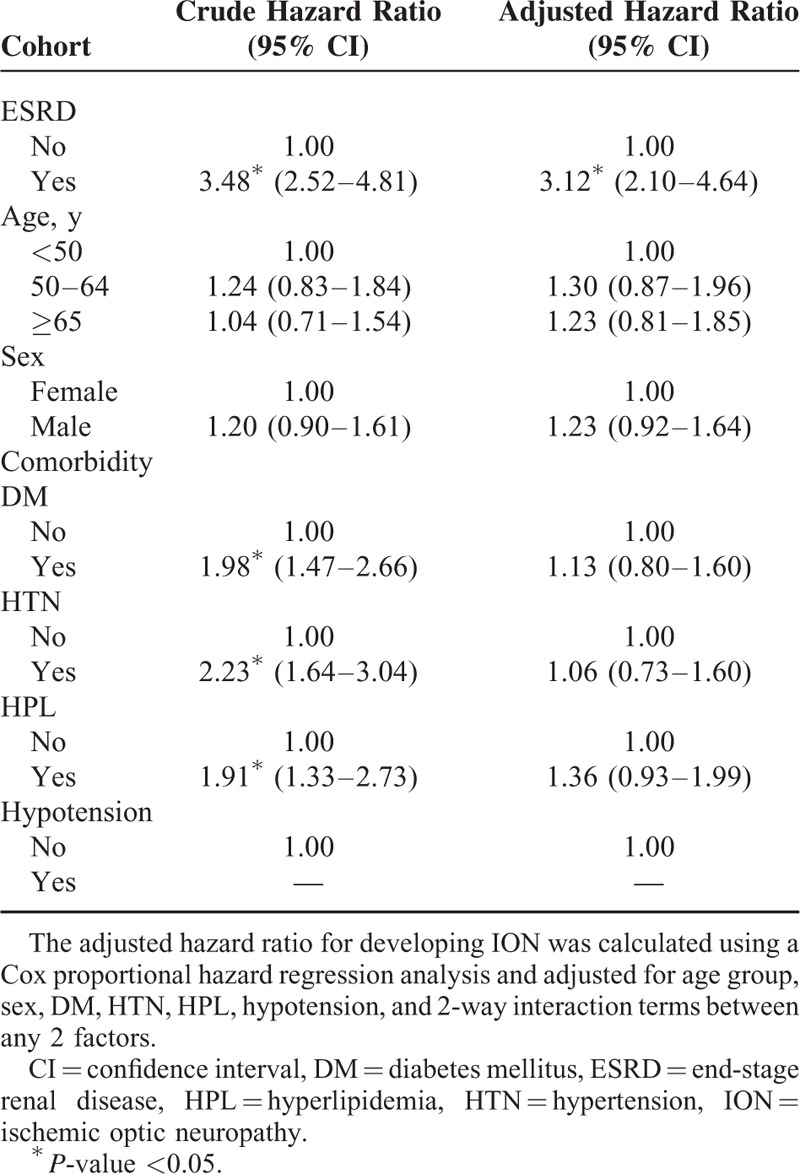
Crude and Adjusted Hazard Ratios of Cox Proportional Hazard Regressions and 95% Confidence Intervals for Ischemic Optic Neuropathy During the Follow-Up period in the Study Cohort

**FIGURE 1:**
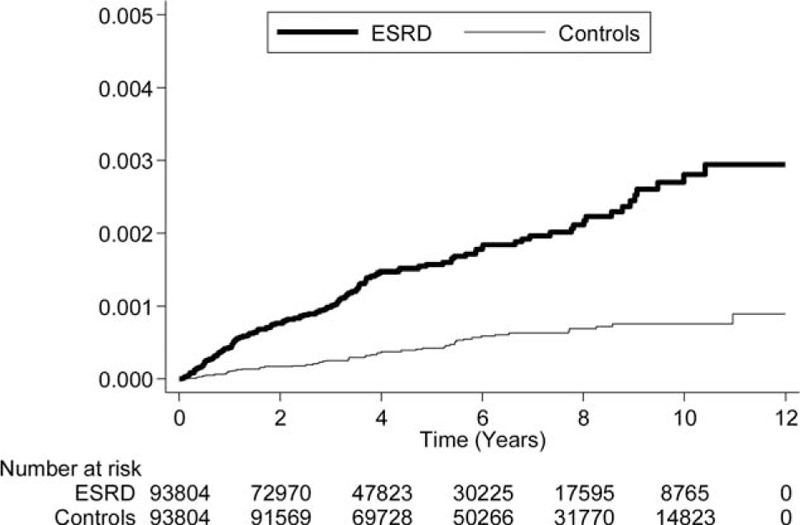
A Kaplan–Meier curve of the cumulative incidence of nonarteritic anterior ischemic optic neuropathy (NAION) in patients with end-stage renal disease (ESRD) and the controls during the follow-up period.

Kaplan–Meier survival analyses revealed higher NAION cumulative incidence rates in ESRD patients than in control patients, and the log-rank test was also significant (*P* < 0.001; Figure 1).

## DISCUSSION

To the best of our knowledge, our study is the largest scale population-based study conducted to explore the relationship between ESRD and subsequent NAION. We analyzed 93,804 ESRD patients and 93,804 control subjects. We found that the incidence rate of NAION in ESRD patients was 3.14 times higher than that in controls, and that the relative risk of NAION in patients with ESRD was increased by 3.12 times in the full cohort after adjusting for age, sex, diabetes mellitus, hypertension, hypotension, hyperlipidemia, and 2-way interaction terms between any 2 factors.

NAION is the most frequent acute optic neuropathy observed in elderly populations and indicates hypoperfusion and ischemia of the anterior portion of the optic nerve.^7,25^ The pathophysiology is multifactorial, besides the well-known small and crowded disc, other contributing factors may include systemic hypotension, acute bleeding with anemia, atherosclerosis, and general atherosclerotic disease such as hypertension, diabetes mellitus, hyperlipidemia,^6,9,26^ and hypercoagulability.^7,11,27^ The association between renal dysfunction and NAION has been explored previously in several studies, but they are limited to a single case or small case series.^28–31^ Servilla and Groggel^28^ first reported a long-term hemodialysis patient that developed severe vision loss after dialysis-induced hypotension episodes and described the link between ESRD and NAION. Haider et al^29^ observed 60 renal failure patients on dialysis and found that four patients developed typical NAION due to hypotension, severe anemia, and generalized atherosclerosis. Besides hemodialysis, Jackson et al^30^ reported that a patient undergoing continuous ambulatory peritoneal dialysis developed NAION because of systemic hypotension. Less commonly, children on chronic dialysis have been reported as prone to develop NAION in several case reports.^31^ Our study is the largest nationwide, population-based cohort study to investigate the risk of NAION following ESRD in Taiwan.

Our findings demonstrate an association between NAION and ESRD. Several common pathogenic mechanisms of NAION and ESRD, including decreased blood delivery, low blood oxygen carrying capacity, and increased vascular resistance, will be discussed separately below.

The most well-known pathogenic mechanism common to both conditions is decreased blood delivery. Intradialytic hypotension is a serious complication affecting ESRD in hemodialysis patients. It occurs when normal cardiovascular responses, such as vascular refill from interstitial tissue, increases in heart rate and contractility, and arterial and venous vasoconstriction, cannot compensate for the decrease in blood volume associated with ultrafiltration.^13^ When hypotension results in hypovolemia, ESRD patients are susceptible to NAION associated with hypoperfusion of the optic nerve and reduced blood flow in the posterior ciliary artery.^10,30,32^ The use of blood volume monitoring, a dialysate cool in temperature, the adequate prescription and frequency of dialysis treatments, ultrafiltration profiling, automatic biofeedback-controlled dialysis, and the administration of arginine vasopressin are advocated as tools to reduce hypotension and therefore prevent subsequent NAION.^14–16^

Another common pathogenic mechanism of both NAION and ESRD is low blood oxygen carrying capacity. ESRD patients generally suffer from anemia, and especially Taiwanese ESRD patients, who have an anemia prevalence of 92.5%.^4,20^ The pathophysiology of anemia in ESRD is multifactorial and includes deficiency in endogenous erythropoietin, iron or folic acid deficiency, shortened red blood cell survival, bleeding tendency, and nutritional inadequacies.^17–19^ Anemia, the leading cause of low blood oxygen carrying capacity, is associated with the development of NAION through resulting hypoperfusion or nonperfusion of the optic nerve head.^6,7,29^ ESRD patients suffering from anemia require treatment with iron supplementation and erythropoiesis-stimulating agents to correct the anemic condition^17–20,33^ and prevent the development of NAION.

Increased vascular resistance from atherosclerosis and hypercoagulable states is the important shared pathophysiologic cause of ESRD and NAION.^9,11^ Many studies have demonstrated that atherosclerosis is common in ESRD populations.^21,22^ The posterior ciliary arteries, which form arterial anastomotic plexuses that supply the optic nerve, originate from the ophthalmic artery.^6,7^ If the arterial blood supply of the posterior ciliary arteries is compromised by general atherosclerosis in an ESRD population, NAION will be prone to develop.^34^ Of particular note is that atherosclerosis in ESRD is usually accompanied and aggravated by systemic generalized atherosclerotic diseases, such as hypertension and hyperlipidemia. For these patients, good control of these systemic disorders is important to prevent further vessel atherosclerosis and the development of subsequent NAION.

Another contributor to increased vascular resistance is a hypercoagulable state. The risk of hypercoagulation disorders is increased in ESRD patients, and possibly related to dysregulation of the coagulation cascade, platelets, and vessel wall due to uremic toxins and metabolic compounds accumulating during renal insufficiency.^23,24^ Besides, many acquired and inherited factors, such as deficiencies in protein C and S and hyperhomocysteinemia, also play important roles in hypercoagulation in ESRD.^35,36^ It is worthy to note that 23% of NAION patients are younger than 50 years,^37^ and that hypercoagulability is a well-known risk factor for NAION in this population.^38^ Many studies have shown that increased blood viscosity, protein C or S deficiency, and hyperhomocysteinemia may precipitate NAION in young patients.^39,40^ Therefore, the hypercoagulation commonly observed in ESRD may participate in the development of NAION, especially in young patients.

NAION is a common and vision-threatening optic nerve disorder in the elderly population. Many comorbidities have been associated with NAION, including hypertension, hypotension, diabetes mellitus, and hyperlipidemia.^9,26^ In this cohort study, we evaluated these comorbidities in ESRD patients and controls and found that hypertension and hyperlipidemia were associated with significantly higher incidences of NAION in ESRD patients than in controls (Table 2). Hypertension and dyslipidemia are well-known causes of atherosclerosis that contribute to the development of NAION. In addition, several studies disclosed that patients with ESRD and hypertension or hyperlipidemia exhibited accelerated carotid atherosclerosis,^22,41^ which is the leading cause of increased vascular resistance and consequent NAION. ESRD patients with hypertension or hyperlipidemia should be advised to control their blood pressure or dyslipidemia because of the significant associations of these diseases with subsequent NAION.

NAION in ESRD is an interdisciplinary emergency, and close collaboration between nephrologists and ophthalmologists is essential. Nephrologists should be aware of the potentially blinding nature of the disease, which typically presents as sudden and painless visual loss, especially on awakening, in ESRD patients on chronic dialysis. The 15% to 20% involvement of the fellow eye in 5 years should be noted by nephrologists and ophthalmologists. The most important concerns for the ophthalmologist are distinguishing NAION from other causes of optic neuropathy such as arteritic, inflammatory, uremic, and compressive optic neuropathy, and other causes of sudden, painless vision loss such as retinal vessel occlusion disease. Although multiple therapies have been attempted, there is no well-established treatment for NAION.^42–45^ Intravitreal injections of glucocorticoids or antivascular endothelial growth factor agents are effective in reducing disc edema, but not in visual outcome improvements.^42^ A large, noncontrolled, retrospective study demonstrated that oral glucocorticoids had an effect on visual outcome improvements.^44^ To avoid glucocorticoid complications, Lee and Biousse^45^ reported that therapy should be considered in NAION patients who have persistent disc edema, bilateral involvement, or unusual progressive vision worsening. Once the diagnosis of NAION is confirmed by an ophthalmologist, the prevention of severe hypotensive events, correction of severe anemia, control of general atherosclerotic disease, and improvement of hypercoagulation status in ESRD dialysis patients are of utmost importance for nephrologists. When managing NAION in ESRD dialysis patients, close cooperation between nephrologists and ophthalmologists is important in this interdisciplinary emergency, and reduces the risk of fellow eye involvement.

There are several strengths to our study. The study performed a highly precise risk appraisal with appropriate statistical power because the study was based on a nationwide and population-based dataset including a large sample of ESRD patients. Additionally, visual disturbance patients visit an ophthalmologist rather than a general practitioner, which reduces selection bias at referral centers and the chances of misdiagnosis. Furthermore, the present study was a cohort study monitoring NAION incidence in ESRD and comparison cohorts with longitudinal data for up to 10 years. Finally, our results are reliable because hypertension, diabetes mellitus, and hyperlipidemia were taken into account as confounding factors when adjusting the HR for NAION in ESRD patients.

There are some limitations to our study. The ICD-9-CM code 377.41 identifies ischemic optic neuropathy (ION), which is classified into anterior or posterior ION and further categorized into nonarteritic or arteritic ION. The patients selected in our study suffered from nonarteritic ION because we excluded patients with arteritic ION by excluding giant cell arteritis (ICD-9-CM code 446.5) from the ION patients (ICD-9-CM code 377.41). However, we could not distinguish nonarteritic anterior ION (NAION) from nonarteritic posterior ION based on the ICD-9-CM code. Most included patients suffered from NAION, as the relative frequencies of anterior ION and posterior ION were 96% and 4%, respectively, in Hayreh report.^7^ Besides, as the sampled patients’ medical histories can only be traced back to the year 1996, we cannot confirm that the controls had no ESRD history before January 1996; therefore, our findings could be compromised. Additionally, several important confounding factors including smoking history, alcohol consumption, medical utilization, body mass index, and small optic disc could not be accessed. Furthermore, some bias may have been introduced because the insurance claims data did not include laboratory data on blood sugar or serum cholesterol levels, or current blood pressure. We considered hypertension, hypotension, diabetes mellitus, and hyperlipidemia as confounding factors to reduce this problem. Finally, the diagnoses of ESRD, NAION, and other comorbid disorders relied on ICD-9-codes, which may have led to disease misclassification.

In summary, our study showed that after adjusting for age, sex, and diabetes mellitus, hypertension, and hyperlipidemia status, ESRD patients showed a significantly higher risk of developing NAION during the follow-up period than the controls. The association between ESRD and NAION is possibly based on the common manifestation of decreased blood delivery, low blood oxygen carrying capacity, or increased vascular resistance. Additionally, ESRD patients with hypertension or hyperlipidemia showed higher incidence rates of NAION. We recommend that ophthalmologists educate ESRD patients regarding NAION, and that nephrologists be aware of the link between hypotension, anemia, and atherosclerosis in an interdisciplinary emergency. Close cooperation between nephrologists and ophthalmologists is necessary to manage NAION following ESRD and reduce the involvement risk of the fellow eye.
